# Epidemiological burden of *Listeria monocytogenes* in Iran

**DOI:** 10.22038/IJBMS.2018.28823.6969

**Published:** 2018-08

**Authors:** Abed Zahedi Bialvaei, Vajihe Sheikhalizadeh, Ali Mojtahedi, Gholamreza Irajian

**Affiliations:** 1 Department of Microbiology, School of Medicine, Iran University of Medical Sciences, Tehran, Iran; 2 Department of Medical Microbiology, Faculty of Medicine, Tabriz University of Medical Sciences, Tabriz, Iran; 3 Department of Microbiology, Faculty of Medicine, Guilan University of Medical Sciences, Rasht, Iran

**Keywords:** Epidemiology, Food, Iran, Listeria monocytogenes, Listeriosis

## Abstract

**Objective(s):**

*Listeria monocytogenes* is a foodborne pathogenic bacteria causing the infection listeriosis, which possibly affects all people, particularly immunocompromised persons and pregnant women. This microorganism can be found in several processed foods, dairy products, raw milk, meat and fish products, seafoods, eggs, fruits, and vegetables. This review discusses about the epidemiological significance, incidence, contamination routes of *L. monocytogenes* in different products and current data about listeriosis in the Iran.

**Materials and Methods:**

For accessing to relevant articles and studies, a search was done in main databases and also, almost all Iranian published articles were studied in this field.

**Results:**

Outbreaks of listeriosis have been reported in many parts of the worldwide, however there is scanty data about the prevalence of listeriosis in Iran. Accordingly, as a result of high incidence of *L. monocytogenes* in women with bad obstetric history or history of abortions, diagnosis procedures for detection of *L. monocyto*genes and timely treatment was suggested.

**Conclusion:**

In spite of low incidence of infection in the past, increased interest for lightly preserved and/or ready-to-eat (RTE) food products has recently led to increasing of *L. monocytogenes* prevalence which has become a public health concern. Subsequently, further researches about the prevalence of *L. monocytogenes* and also antibiotic susceptibility testing is needed to enable the detection of the contaminated foods, as well as ensures the effective treatment.

## Introduction

Listeriosis is a bacterial infection caused by the *Listeria monocytogenes, *which was first reported by Nyfeldt in 1929 (1). Several years later, during the 1980s, the increased number of listeriosis cases in different communities turned into a recognized foodborne disease(2). Listeriosis frequently leads to admission to intensive-care units (ICUs) that makes *L. monocytogenes* the third most costly foodborne pathogenic bacteria in the United States, after *Vibrio vulnificus* and *Clostridium botulinum* in 2010 (3). Due to high mortality rate, listeriosis and outbreaks caused by *L. monocytogenes* have a significant economic effect on public health and the food industries (4). Listeriosis resulted in 172823 disability-adjusted life-years (DALYs), 23150 illnesses and 5463 (23.6%) deaths in 2010 worldwide, as indicated by World Health Organization (WHO) researchers published in The Lancet Infectious Diseases (5). Also, in the European Union (EU), 2194 confirmed cases were reported in 2014 with a rate of 0.6 per 100000 population and a predominance of cases among detected in elderly people over 64 years of age (1.9 per 100 000 population) and among infants below one year of age (2.8 per 100 000 population) (6). Moreover, the food industries as well as regulatory organizations performs a large number of tests for *L. monocytogenes* and Listeria spp., on food and environmental samples; subsequently, detection of Listeria species is frequently utilized by the food industry as a marker to detect conditions that allow for the presence, growth, and persistence of *L. monocytogenes* and have significant effects on test kit manufacturers and food industry. 

Only a couple of nations have evaluated the listeriosis burden in terms of DALYs (5), and the worldwide burden of listeriosis has never been assessed. In any case, DALYs can be used to compare health conditions and diseases, and therefore help policy makers to allocate resources. Iran is among the foremost consumers of dairy based foods and animal sources and therefore the increasing use of antimicrobials in animals has caused the antibiotic resistance problem as a crucial public health challenge. At present, there is a little data with respect to the prevalence of Listeria spp. in the region. Thus, the present review was done to gather the prevalence of *L. monocytogenes* and listeriosis in Iran (7, 8). 

**Characteristics and importance of Listeria**

Listeriosis has become as a serious public health challenge owing to the severity of the disease (meningitis, septicemia and abortion), the high mortality rate, the long incubation period, and the predilection for persons who have an underlying disease (9). The individuals, specially affected by listeriosis are elderly people, pregnant women and their fetus as well as patients with malignant disease, cirrhosis, diabetes mellitus, chronic kidney disease, rheumatoid arthritis, collagen-vascular diseases, and alcoholism (10). Moreover, *L. monocytogenes* in spite of most other foodborne pathogens, grows in food with high salt concentration and fairly low moisture content. Most significantly, this pathogen can grow at refrigeration temperatures. Furthermore, they have been found on the body surface and in the animal intestinal tracts, and have been isolated from the livestock feces and farm drainage (11, 12). Because of persistence and multiply in the food environment, makes *L. monocytogenes *of high concern to the food industries and is difficult to control (13). 

Basically, there are two types of invasive and noninvasive listeriosis. Noninvasive form is related with a huge intake of bacteria (14). *L. monocytogenes* can be transmitted through utero/parental transmission or from person to person. But, the greater part of instances of human listeriosis are foodborne (15). The noninvasive listeriosis is generally seen in healthy adults, in whom *L. monocytogenes* does not cross the gastrointestinal (GI) barrier. In the invasive form, *L. monocytogenes *invade the mesenteric lymph nodes and reach the bloodstream. Despite of its low incidence, this form has created great concern to public health due to its severe symptoms, life-long consequences and high fatality. The invasive listeriosis has three major clinical presentation; bacteraemia, central nervous system (CNS) infection and pregnancy associated listeriosis. Furthermore, a variety of focal infections are also described (16). Human Listeriosis is mostly identiﬁed as invasive forms. 

***Bacteraemia***

Invasive listeriosis generally presents as bacteraemia with or without an obvious focus of infection (17). Clinical picture of bacteraemia because of *L. monocytogenes* are same with other etiological agents, present as acute febrile disease and frequently accompanied by myalgias, arthralgias, backache and headache. The infection may advance to acute respiratory distress syndrome, disseminated intravascular coagulation and multi-organ system failure (18, 19). In 2014, a total of 660 cases of invasive listeriosis were reported from USA patients, of which 459 (69.5%) were bloodstream infections and led to death in 107 (23.3%) of inhabitants. Early bacteraemia was also the most common clinical feature of *L. monocytogenes* in an investigation from France between 2009 and 2013, with frequency of 52.2% and mortality rate of 45% (18). The median age of the patients was 73 years and they were found to had at least one of the following symptoms: fever, inﬂuenza-like symptoms, decompensated comorbidity, diarrhea, and multi-organ failure (20). Similarly, 90% of listerial bacteraemic patients in England and Wales were reported to have underlying conditions. Likewise, speciﬁc malignancies (especially digestive-organ malignancies) were more prevalent among the bacteraemic patients (21). 

***Central nervous system infections***

A characteristic feature of listerial infections is the central nervous system (CNS) involvement and accounts for 30.7% of all non-perinatal listeriosis cases (5). CNS infection with *L. monocytogenes* manifests most commonly as brain abscess, meningitis, cerebritis rhomb encephalitis, and spinal cord abscess (22). Detected risk factors for severe Listeria infections, which include Listeria encephalitis, were senility, malignant hemopathies, cancers, immunodeficiency, chronic liver disease, chronic hemodialysis, and alcohol poisoning (23). In spite of the fact that the exact mechanism(s) utilized by *L. monocytogenes* for CNS entering are not clear, current hypothesis demonstrates that neuroinvasive bacteria in overall can enter the CNS by a few distinct ways (24). These include invasion of microvascular endothelial cells, invasion of epithelial cells of the choroid plexus, and passage of bacteria through intercellular intersections. Moreover, bacteria that are able to intracellular survival, can enter the CNS by means of phagocyte-facilitated infection, the main steps of which are attachment of infected phagocytes to endothelium took after by cell-to cell dissemination of bacteria to endothelial cells and/or migration of infected phagocytes into the CNS (25). A meta-analysis assessed that 31% of listeriosis cases were neurolisteriosis patients worldwide (5). In another study, rhomb encephalitis (58%) was the most clinical sign of *L. monocytogen*es encephalitis (26). *L. monocytogenes* was the fourth identified cause of encephalitis in metropolitan France, yet represented 5% of cases (26, 27). Listeria encephalitis may be classified as meningitis in different nations. In addition, meningitis and meningoencephalitis are the most clinical presentations of CNS infections accounting for 70-97% of cases (28, 29). 

***Pregnancy-related listeriosis***

Compared to the general population, pregnant females have 10 times higher risk for listeriosis (30). In about 29% of cases, the mother infection might be asymptomatic or represented as a flu-like disease with headache, fever or myalgia. However, it may has sever outcomes, including miscarriage, stillbirth, and prematurity delivery (31). Of these, premature birth, with the occurrence rate of 64% is a frequent complication of listeriosis in the pregnant women (30). About 15% and 6- 18% of listeriosis cases in USA, and Europe, respectively, are related to pregnancy (20, 32-34).

Neonates usually acquire the infection through the transplancental transmission from maternal bacteraemia or during delivery through the vaginal canal. Moreover, ascending infection from the lower reproductive tract of mother is associated to be other possible route of infection (35). Some of the complications of listeriosis in newborns include: physical retardation, granulomatosis infantiseptica or death. In fact, neonatal listeriosis is subdivided into early-onset and late onset diseases. Early onset illness (granulomatosis infantiseptica) is often overt within 7 days after birth and accounts for the majority of neonatal cases. Infants with early onset illness acquire infection in-utero from the bacteraemic mothers and are frequently born preterm. Bacteraemia (81–88%), pneumonia or respiratory distress (38%) and meningitis (24%) are the common presentations of this infection (36). Moreover, meconium staining, lethargy, fever and jaundice may be observed. Less commonly, approximately in 5-31% of cases, late onset neonatal listeriosis can occur with mean onset age of 14 days after birth (20, 33). This form of infection occurs more frequently in full term neonates born from the asymptomatic mothers. 

***Febrile gastroenteritis***

In healthy people, *L. monocytogenes* can cause a mild and self-limiting gastroenteritis. Listerial gastroenteritis is differentiated from invasive listeriosis with aspects of incubation period, symptoms and mortality. It usually occurs in healthy adults following ingestion of high doses of the organism (37). Following 6–49 hr incubation period, the illness represented as diarrhea, fever, chills, abdominal pain, myalgias and headache (38, 39). This is a self-limited illness with median durations of 42 hr and most patients recover without antimicrobial therapy (38).

***Localized infections***

*L. monocytogenes* can also cause a variety of localized infections. The infrequent local infections as the consequent of Listerial bacteraemia include peritonitis, endocarditis, splenic abscesses, cholecystitis, hepatitis, endophthalmitis and osteomyelitis (18). Direct inoculation of *L. monocytogenes* may also prompt cutaneous and conjunctivitis infections (40).

**Listeria species and food products**

Foodborne diseases show a growing health issue across the globe and more than two hundred different diseases are known to be transmitted by food (41). Most authors mentioned that 99% of the human listeriosis have a foodborne origin (42). *L. monocytogenes* has been isolated from many foods, for example milk, and ice cream; meat and meat products, vegetables; fish products; and different Ready-to-Eat (RTE) foods (42). There are recent investigations on *L. monocytogenes* that demonstrate the presence of this bacterium in a few foods and RTE products (42, 43). Additionally, certain foods have been described as “high risk” for listeriosis. 

There are no comprehensive recommendation or criteria for foodborne *L. monocytogenes* in Iran. The eating habits of Iranian population are also unique in relation to those of Western nations. Different locally produced and traditional foods are consumed in Iran and industrial stuff knowledge about prevalence importance of *Listeria *in food is fundamental. The initial step to persuade private industry and regulatory authorities about the importance of Listeria in foods is to give a data on prevalence of bacterium in different foods. Type of food tested and their rate of contamination with Listeria spp. in Iran are shown in Table 1, 2 and 3.

***Dairy products***

The importance of raw milk and dairy products as a vehicle for the transmission of several diseases has been reported; particularly in nations where hygienic standards are not strictly performed. Milk and dairy products are two particular food categories concerning the risk evaluation for listeriosis. The most vital risk factors of *L. monocytogenes* contamination of raw milk includes lack of correct management of barn and silage, insufficient hygiene practice in the environment, defective disinfection of teats before milking, and a low level of cleanliness among cows (44). Hygienic control of milking and milking system are considerations that have a significant statistically association with risk of Listeria persistent in bulk tank milk (45). Hence, exogenous contamination of milk with fecal material because of bad practices of hygiene standards is the most important concern during milking. There are also challenges of good practice during transportation and storage of milk that require consideration (44, 45). In a study by Rahimi *et al* carried out in Isfahan, Iran (46), among all the different tested milk and dairy products, raw milk samples and traditional cheese (made from raw milk of cow or sheep) had the highest prevalence rate of Listeria species. In spite of the fact that the prevalence of Listeria spp. may vary in various dairy products, it has been demonstrated that Listeria isolates can be detected more frequently in soft cheeses and raw milk samples (46). To determine the level of listeria contamination from dairy products sent to the Lorestan laboratory of the Food and Hygiene, a study was conducted by Mojtahedi and his colleagues in 2005. In this study, *L. monocytogenes* in 9.72% of samples and *L. innocua* and *L. seeligeri* were found in 5.83% and 1.11% of samples, respectively. Other two studies from Isfahan and Kurdistan provinces (47, 48) reported that about 6% of raw milk samples contaminated with Listeria species. In Isfahan study, four of five isolates identified as *L. monocytogenes* and one as *L. seeligeri*. Further, a similar study showed that utilization of raw milk with mild heat treatment or its usage in conventional dishes is a typical practice in Iran (49). This study was carried out on 292 samples of traditional, unpasteurized and raw milk dairy products showed that 4 (1.36%) and 21 (7.19%) were positive for pathogenic *L. monocytogenes* and Listeria spp., respectively. This study showed that the prevalence of Listeria spp. in ice cream, cream, raw milk, and freni was 12/63 (19.04%), 3/27 (11.11%), 5/91 (5.49%), and 1/25 (4%), respectively (49). The general quality of food samples and marketplace sanitation contamination with Listeria spp. in Iran are shown in Table 4.

Traditional Lighvan cheese has a popular market in Iran and neighbors (50). This is a semi-hard cheese, which produced from raw sheep’s milk or a mixture of raw goat and sheep milks without added starter in Lighvan region of East Azarbaijan province (Northwest of Iran). Annually, at this region, nearly 3150 tons of Lighvan cheese is produced (50). The ideal flavor of Lighvan cheese is ascribed to its natural flora (51). Because the Lighvan cheese is created from unpasteurized raw milk and there’s no heating method during its production (50), pathogenic bacteria may also survive and grow with this decline in these cheeses. Although there is a considerable evidence about the contamination of milks and dairy products by *L. monocytogenes* around the world, there are few data about the contamination of Lighvan cheese and milks that are used for its production (Table 2). Putting unwrapped raw materials in refrigerators might enable these organisms to enter the refrigerators and pose a health risk in the kitchen (52). Kargar *et al.* (53) indicated a notable contamination of fresh cheese with *L. monocytogenes* in Marvdasht, Iran. In this study, *L. monocytogenes* was isolated from 56 samples (%13.08). Fisher test indicated significant correlation between months of sampling and *L. monocytogenes *isolation (*P*=0.004). In addition, all the isolates was sensitive to ampicillin and most resistance was attributed to tetracycline and ceftriaxone.

**Table 1:** Type of raw materials tested and their rate of contamination with Listeria spp. in Iran

**Type of foods**	**Year**	**City**	**Total** **samples**	**Contamination with** ***Listeria *** **spp.**	**Contamination with ** ***L.*** ***monocytogenes***	***reference***
			**No.**	**No. (%)**	**No. (%)**	
***Raw***						
Chicken	2012	central part	54	22 (40.7)	9 (17.6)	(70)
Turkey	2012	central part	40	10 (25.0)	5 (12.5)	(70)
Quail	2012	central part	33	7 (21.2)	3 (9.10)	(70)
Ostrich	2012	central part	21	1 (4.67)	0(0.0)	(70)
Chicken liver	2012	central part	51	29 (56.9)	11 (21.6)	(70)
Salt water fish	2012	Ahvaz	70	ND^a^	1 (1.4)	(65)
Shrimp	2012	Ahvaz	70	ND	1 (1.4)	(65)
Fish and shrimp	2012	Isfahan and Shahrekord	264	20 (7.5)	5 (1.9)	(52)
freshwater fish	2013	Central part	105	ND	12 (11.4)	(57)
seawater fish	2013	Central part	167	ND	3 (1.8)	(57)
seawater shrimp	2013	Central part	59	ND	1 (1.69)	(57)
Fish	2013	Isfahan and Shahrekord	220	23 (10.45)	17 (7.72)	(66)
Shrimp	2013	Isfahan and Shahrekord	40	1 (2.5)	1 (2.5)	(66)

a ND: not defined

**Table 2:** Type of dairy products tested and their rate of contamination with Listeria spp. in Iran

**Type of foods**	**Year**	**City**	**Total** **samples**	**Contamination with** ***Listeria *** **spp.**	**Contamination with ** ***L.*** ***monocytogenes***	***reference***
			**No.**	**No. (%)**	**No. (%)**	
***Dairy products***						
Milk	2007	Shahrekord	500	11(2.2)	8 (1.6)	(108)
Cheese	2010	Isfahan	90	17 (18.9)	9 (10.0)	(44)
Ice cream	2010	Isfahan	68	7 (10.3)	2 (2.9)	(44)
Yogurt	2010	Isfahan	35	0(0.0)	0(0.0)	(44)
Doogh^a^	2010	Isfahan	30	0(0.0)	0(0.0)	(44)
Butter	2010	Isfahan	40	2 (5.0)	1 (2.5)	(44)
Milk	2011	Kerman	100	ND^b^	5 (5.0)	(42)
Ice cream	2013	Kermanshah	67	1 (1.5)	0(0.0)	(109)
Milk	2013	Kermanshah	59	6 (10.2)	0(0.0)	(109)
Milk	2013	Shahrekord	596	ND	58 (9.73)	(110)
Cheese	2013	Kermanshah	59	0(0.0)	0(0.0)	(109)
Milk	2014	Tabriz	18	ND	9 (50)	(111)
Cheese	2014	Tehran	70	ND	2(2.85)	(112)
Cream	2014	Tehran	5	ND	0(0.0)	(112)
Kashk^c^	2014	Tehran	5	ND	0(0.0)	(112)
Cheese	2015	Tehran	70	ND	5(1.7)	(113)
Cream	2015	Tehran	20	ND	3(15)	(113)
Curd	2015	Tehran	17	ND	3(6.1)	(113)

a A dairy product prepared by beating unflavored yogurt until smooth, and then diluting with water to a consistency similar to whole milk; it is also called yogurt soda;

b ND: not defined;

c A dairy product prepared by prolonged boiling yogurt

**Table 3:** Type of meat, ready-to-cook and ready-to-eat products tested and their rate of contamination with Listeria spp. in Iran

**Type of foods**	**Year**	**City**	**Total** **samples**	**Contamination with** ***Listeria *** **spp.**	**Contamination with ** ***L.*** ***monocytogenes***	***reference***
			**No.**	**No. (%)**	**No. (%)**	
***Meat products***						
Kielbasa^a^	2013	Kermanshah	58	4 (6.9)	2 (3.5)	(109)
Sausages	2013	Kermanshah	56	3(5.4)	0(0.0)	(109)
Minced meat	2013	Kermanshah	73	44 (60.3)	1 (1.4)	(109)
Different kind of meats	2013	Tehran	410	ND^b^	115 (28.05)	(114)
Sausage	2014	Tehran	30	ND	0(0.0)	(112)
Chicken concentrate	2014	Tehran	10	ND	1(10.0)	(112)
Calf meat concentrate	2014	Tehran	10	ND	1(10.0)	(112)
***Ready-to-cook***						
Barbecued	2012	central part	45	25 (55.5)	9 (20.0)	(70)
Chicken	2012	central part	42	8 (19.0)	3 (7.14)	(70)
Chicken nugget	2012	central part	28	5 (17.9)	2 (7.14)	(70)
***Ready-to-eat***						
Oloveyh salad^c^	2007	Isfahan	30	2(6.6)	3(10)	(69)
Olovieh salad	2012	central part	32	25 (78.1)	10 (31.2)	(70)
Chicken	2012	central part	26	1 (3.85)	0(0.0)	(70)
Chicken burger	2012	central part	30	1 (3.33)	0(0.0)	(70)
Olovieh salad	2013	Kermanshah	11	2 (18.2)	0(0.0)	(109)
Fruit juice	2013	Kermanshah	55	1(1.8)	0(0.0)	(109)
Green salad	2013	Kermanshah	92	5 (5.4)	0(0.0)	(109)
Fish and shrimp nugget	2016	Karaj and Tehran	79	8 (10.12%)	0(0.0)	(67)

a Type of sausage, usually served uncooked;

b ND: not defined;

c Mayonnaise-based salad containing cooked chicken meat, potatoes, sour cucumber and green beans

**Table 4:** General quality of food samples and marketplace sanitation contamination with Listeria spp.

**Variable**	**No. of** **samples**	**Contamination with** ***Listeria *** **spp.**	
		**NO.**	**%**
***Food quality score***			
** 1**	12	6	50.0
** 2**	99	14	14.1
** 3**	184	22	12.0
**4**	157	18	11.5
**5**	78	6	7.7
**Marketplace sanitation score**			
**1**	31	7	22.6
**2**	66	8	12.1
** 3**	188	22	11.7
** 4**	193	23	11.9
** 5**	52	6	11.5
***Total***	530	66	12.5

Samples are classified according to the general conditions of cleanness and sanitation of the market outlet (scoring from 1 to 5 score representing bad to excellent conditions). Data are adapted from Akya *et al.* (110)

***Seafood products***

Listeria spp. has been isolated from an extensive variety of seafood products like crab (8), shrimp (54), fish products (55), cold-smoked rainbow trout (56) and lobster (57). In overall, seafood product captured from the contaminated waters may possibly convey *L. monocytogenes*. Also, they can be contaminated during transportation and in the fish marketplace (58). In seafood processing industries, transient *L. monocytogenes* from raw materials might contaminate the final products. On the other hand, it is determined that persistent in-house strains of *L. monocytogenes* might also be the source of contamination for the final products (59, 60). The bacteria may enter the processing plant by contaminated water, utensils, raw materials, and staffs; therefore contaminating the processing materials and final products (58). In addition, light preservation processes like marinating, cold-smoking and curing may not be sufficient to eliminate *L. monocytogenes* that might be present on raw materials (59, 61). Although *L. monocytogenes* has been isolated from fish, and seafoods, no major outbreaks of listeriosis due to these products has been reported yet (62). But, these contaminated products are considered as the most prevalent causes of a number of sporadic listeriosis cases (63). 

Seafood products are so prevalent within Iranian individuals and the utilization of seafood products has increased recently as a result of increased consumer awareness about nutrition and food quality. Nevertheless, in spite of the high importance of seafoods listeriosis, there were few published data about its distribution in fish and shrimp samples of Iran (Table 1). According to the results of these studies, the Listeria spp. had the low frequency in Iranian seafoods. Basti *et al.* (2006) showed that 2.6% of smoked fish samples in Gilan province were positive for *L. monocytogenes* (64). While, another investigation by Modaresi (2011) revealed that 12.37% of collected fish samples from Urmia fish markets were positive for Listeria spp. (65). They reported that 29% and 21% of isolates were *L. ivonoi *and *L. monocytogenes, *respectively. Also, the low frequency of *L. monocytogenes* (about 1.6%) was reported by other studies in 2012 (8, 66). In a survey, 300 different seafood samples were collected from the retailers and supermarkets of Shahrekord and Isfahan cities in 2013 (67). The results of this study showed that 0.66%, 0.66%, 1%, and 6% of Iranian seafood samples were positive for *L. innocua*, *L.* *seeligeri*, *L. ivanovii*, and *L. monocytogenes*, respectively (67). In another survey by Abdollahzadeh *et al.* (2016), the prevalence of Listeria spp. was investigated in a total of 237 fish, shrimp, processing plant and ready-to-eat seafood samples at Karaj and Tehran, Iran (68). In this survey, 8.86% of the total processing plant and retail samples were positive for Listeria spp., which 7 (2.95%) of the total samples were also positive for *L. monocytogenes*. Moreover, four virulence-associated determinants (*inlA, inlC, inlJ*, and *hlyA*) were detected in six fish isolates. 

Based on the data presented in this review article, hazard analysis and critical control points (HACCP), or alternative food safety programs plus routine management of contamination with *L. monocytogenes*, should be enforced in food facilities to manage and reduce the potential risk of *L. monocytogenes*. The national HACCP committee has been set organized in order to expand HACCP throughout the food industry. To use HACCP, several food industries meet the sanitary necessities and Good Manufacturing Practice as a prerequisite to HACCP. The Iranian shrimp industry is additionally forced by the European Community (EC) to process under HACCP concepts in order to export their products to Europe. The successful introduction of HACCP in the shrimp industry prompted to pressure for their wider acceptance by the food industry. Despite the fact that an outcome of the implementation of HACCP in Iranian food industry has not been well documented, this may control the presence of Listeria spp. in food in Iran. 

***Chicken products***

People in Iran consume various types of meat, for example, chicken, turkey, ostrich, mutton, beef, camel and quail either fresh or frozen. Nonetheless, chicken, mutton, and beef are favored. Rahimi *et al.* (2008) isolated 3% *L. monocytogenes* from beef carcasses in Isfahan, Iran (69). In another report from this city by Jalali and Abedi (2008), *L. monocytogenes* was identified in 14.2% of frozen beef, 6% of sheep meat and 2.6% of fresh beef (70), whereas in the another survey from Isfahan and Shahrekord cities by Rahimi *et al.* (2012), 12.7% Listeria spp. was isolated from various kinds of raw meat, and 19.1% of them determined as *L. monocytogenes *(8). The prevalence of Listeria spp. in central part of Iran, from a total of 402 popular poultry product samples examined, 134 samples (33.3%) were contaminated with Listeria species, which 52 (12.9%) were *L. monocytogenes* (71). The data demonstrated in this review point out the potential risk of eating raw and undercooked meats in Iran. Since a few people consume raw or undercooked meat in Iran, utilization of them may pose risk of foodborne infections. The prevalence and antibiotic resistance of Listeria spp. isolated from Chicken nuggets was determined in a study from Isfahan province (72). In this survey, the highest rate of infection with Listeria spp. was 8%, which the most antimicrobial resistance was belonged to nalidixic acid (86%) followed by ciprofloxacin (43%).

***RTE***

In recent years, we faced to the growing consumption of RTE foods as the foundation of *L. monocytogenes* outbreaks, at which most RTE foods are stored for quite a while and some of them are warmed for a couple of minutes or not reheated before serving (68). Cases of RTE refrigerated foods include deli salads, soft cheeses, pre-packed fresh vegetables and fruits and seafood salads. The presence of *L. monocytogenes* in these products may result from contaminated raw materials or from cross-contamination throughout packaging, processing, or retail presentation (73). The seafood salads safety can be guaranteed by a mix of preservative use, refrigerated storage, and addition of organic acids to decrease the pH of the final product. Nonetheless, when the contamination involves a resistant/adapted pathogenic bacteria, this food-preservation technique might be insufficient and in this way compromise food safety (74).

**Incidence and prevalence of listeriosis**

Among the recent 20 years, listeriosis has turned into an alarming disease in several countries. At 1997, the prevalence of listeriosis was assessed to be responsible for 500 deaths every year in the US, and in 2000, listeriosis was estimated to be 4 per million population. A marked decrease in pregnancy-associated listeriosis was reported in the US between 2003 and 2007; interestingly, an increase has been reported in Wales and England (110 cases per year between 1990 and 1999 versus 191 cases between 2001 and 2009) (21). In 2014, 675 cases of listeriosis were reported to the *Listeria *initiative surveillance system in the USA which of these, 660 cases (98%) were invasive (32)**.** Overall, it is an uncommon infection; it has been responsible for that the yearly incidence of listeriosis rates changes between 1 and 11.3 per million population with approximately 20% involving neonates (21, 75). In the USA, Centers for disease control and prevention (CDC) estimates 260 deaths resulted by 1600 annual cases of listeriosis (76). 

There is no totally organized data regarding the epidemiology of listeriosis in Iran. Additionally, listeriosis is not a reportable disease within the Iranian health system and there are no criteria for listeriosis in food industries in the country. The first case of the listeriosis in Iran was reported by Nazari *et al.* in 1963, which Listeria spp. isolated from a patient suspected to pulmonary tuberculosis (77). In another early study that conducted during 1965-1971 in Iran, 500 patients with various complications and suspected to Listeria were tested for anti- listeria antibodies (78). 

As mentioned in above,* L. monocytogenes* is an important causes of abortion and postpartum infection in newborns. In 210 cases that had one abortion, listeria antibodies were found only in one (4%) patient, and in 208 cases, which had more than one abortion listeria antibodies were found in 20 (12.5%) cases. Listeria antibodies were not found in the rest of the patients referred due to infertility or acute infectious complications (78). Also, three cases of *L. monocytogenes* were recovered from mothers and infants by Lashgari *et al.* in 1974 (79). In 1988, Vand Yousefi *et al.* also conducted a study on culture and serology testing, and isolated serotypes were 2a, 4a and 4b. Given the fact that they are of a variety of foodborne serotypes, it indicates their importance in food contamination (80). In an investigation carried out in Teheran, children’s sera were tested for Listeria antibodies at 1989 (81). In this study, 1–11% of sera were seropositive against one of several serotypes of Listeria spp. Also, a case–control study was performed in 2009 for evaluating seropositivity for *L. monocytogenes* in women of child-bearing age with spontaneous abortion (82). This study uncovered that 18% of those women with normal full-term deliveries and 36% of those with a background of spontaneous abortion were positive for *L. monocytogenes* antibody (82). In another study, to evaluate the effect of *L. monocytogenes* on pregnant women in 2009, 204 women were chosen randomly and sera were used for exploring Listeria specific antibody by indirect immunofluorescent method (83). The data demonstrated that Listeria has been a causative agent of 12.5% of abortions. In addition, the serologic study results significantly revealed that 25 cases of test group had an antibody titer of > 1/160 against *L. monocytogenes*. Also, during 2009-2010, nine *L. monocytogenes* from 100 clinical samples of patients with spontaneous abortions were tested, which was higher than the earlier reports (84). Out of these isolates, 3 (16.66%), 3 (12%), 2 (8%), 1 (4%), and 0 (0%), *L. monocytogenes* were isolated from placental tissue, vaginal swabs, rectal swabs, urine and blood, respectively. It was mentioned that the differences reported among these studies could be due to differences in the population under investigation including culture, race, nutrition, geographical region and laboratorial diagnostic methods. In a similar study that carried out between 2010-2013, 14 *L. monocytogenes* was recovered from 170 clinical samples of patients with spontaneous abortion hospitalized in Shariati Hospital (85). Out of 14 *L. monocytogenes* isolates, 5 (35.71%), 4 (28.57%), 3 (21.42%), 2 (14.28%), 0 (0%), were isolated from vaginal swabs, placental tissue, rectal swabs, urine and, blood, respectively. As a result, in view of high frequency of *L. monocytogenes* in women with bad obstetric history or history of abortions, diagnosis procedures for detection of *L.* *monocytogenes* and timely treatment was suggested. Furthermore, due to high antimicrobial resistance rate of bacteria, antibacterial susceptibility before initiation of treatment was recommended (84). To detect the prevalence of *L. monocytogenes* in pregnant women and to compare the level of prevalence among women with an abortion history and with no a history of abortion, 540 samples of pregnant women were provided from Arak Taleghani Hospital (86). In this study, 14 cases had *L. monocytogenes*, which eight cases had a history of abortion. 

**Susceptibility status of** ***L. monocytogenes***

Although, the risk of infectious disease has decreased by the use of antimicrobials in poultry production stage, it might prompt the spread of antimicrobial-resistant bacteria as well as resistant strains of Listeria within the environment. The transmission of the resistant bacteria to human by contaminated food products could have public health consequence. Furthermore, excessive utilization of antibiotics in veterinary medicine have leads to the distribution of antimicrobial-resistant strain within the environment (87). Fluoroquinolones and tetracycline are widely used as therapeutic agents and growth supplement in fish farms, respectively. Considering the presence of antibiotic resistant *L. monocytogenes* and additionally multi-drug resistant (MDR) bacteria in fish on the one hand and transmission of the pathogen through contaminated fish on the other hand, clarifies major public health concerns related to this bacteria. According to the data, there is a high resistance of Listeria spp. to penicillin, nalidixic acid, ciprofloxacin and tetracycline, and to a lesser extent to ampicillin, erythromycin, and chloramphenicol (46). Therefore, monitoring the antimicrobial resistance of *L. monocytogenes* in animals and humans has most extreme significance to implement pro-active measures to control the use of antimicrobial agents, identify changes in the patterns of resistance to commonly used antimicrobial agents, and prevent the spread of MDR bacteria. However, in another report, *L. monocytogenes *was highly susceptible to ampicillin (100%) and trimethoprim (100%), streptomycin (85.71%), ciprofloxacin (78.57%), tetracycline (64.28%) and norfloxacin (64.28%), but highly resistant to penicillin G (57.14%) (85).

**Reduction in the incidence of invasive Listeriosis**

Microbiological risk assessment provides an estimate of the probability of illness from a particular pathogen in a given population. In addition, detection of outbreak and investigation have had a basic role in distinguishing particular improvements required to further lower the incidence of listeriosis. Despite the downward trend in *L. monocytogenes* infection related to poultry products and RTE meat, a few large multistate outbreaks of listeriosis caused by RTE foods happened toward the end of the 1990s. As a result of outbreaks related to hot dogs and turkey delicatessen meat during 1998 and 2000 (88), the United States Department of Agriculture- Food Safety and Inspection Service (USDA-FSIS) and Food and Drug Administration (FDA) examined ongoing prevention and control activities for *L. monocytogenes* and developed a Listeria action plan in 2001. The USDA-FSIS presented an interim final rule following a second outbreak associated with turkey delicatessen meat in 2002 (89), that needed governmentally inspected facilities producing certain (RTE) poultry products and meat to take steps to further decrease the incidence of *L. monocytogenes* infection (90). In 2003, the FDA updated the Listeria action plan to reduce listeriosis due to the consumption of RTE foods in the regulatory purview of the FDA (91). The revised Listeria action plan focuses on high-risk foods and includes methods for training, guidance, education, research, enforcement and surveillance. 

From ‘‘what-if scenarios’’ and the exposure models utilized as a part of the risk assessment, it was determined that the following five factors influenced consumer exposure to *L. monocytogenes* at the time of food consumption: (1) frequency and levels of *L. monocytogenes* in ready-to-eat food, (2) frequency and amount of consumption of a food, (3) refrigerated storage temperature, (4) the likelihood of the growth of *L. monocytogenes* in a food during refrigerated storage and (5) duration of refrigerated storage of a food before consumption. The model of risk assessment was utilized to determine the likely effect of control strategies by changing one or two input parameters and estimating the change in the model yields. For instance, one ‘‘what-if’’ scenario discovered that the predicted number of listeriosis cases would be decreased by 69% if all home refrigerators were consistently working at or below 7.2 ºC. Another scenario confirmed that reducing the maximum storage time of deli meats from 28 to 14 days will reduce the median number of cases in the elderly population by 13.6% (37).

Following a listeriosis outbreak related to contaminated ‘‘rillettes’’ (a kind of pate made from pork that has a shelf-life of around 42 days, and is ordinarily removed from the refrigerator and placed back multiple times at home), investigators verified that there was a noteworthy difference in the number of times (6 vs. 4) the food product was moved between the refrigerator and the dining table between case households and control households (37). Additionally, it was determined that an initial contamination level of 1 colony forming unit (CFU) per 100 g would render the product dangerous in 32 days, though an initial contamination level of 10 CFU/g would render the product unsafe within 8 days. Salvat and Fravalo (92) inspected the risk factors at the pig processing and production stages and resulted that live pigs frequently contain *L. monocyotgenes* strains implicated in epidemic outbreaks. Also, it was suggested that prevention efforts should be focused on reducing the contamination on the slaughterhouses and at the farms.

**Characterization methods**

Reports of human listeriosis in Iran are unclear, either on account of losing on recognizing the isolates, unsuitable isolation techniques or absence of awareness. To prevent the contamination of products with food spoilage bacteria and poisoning-bacteria, it is important to determine the routes of contamination and their place of manufacture (93). Phenotypic or genetic characterization through subtyping analysis enables the detection of infection sources (94). Classification based on serotype is valuable for tracking of *L.* *monocytogenes *strains linked to disease outbreaks. *L. monocytogenes *is classified into 13 serotypes (1/2a, 1/2b, 1/2c, 3a, 3b, 3c, 4a, 4ab, 4b, 4c, 4d, 4e, and 7) (95). However, this classification gives restricted discrimination during epidemiological examinations in light of the fact that the dominant part of outbreaks and human listeriosis cases are predominantly associated to 3 serotypes (4b, 1/2b, and 1/2a) (96). Greater outbreaks have been primarily related to the 4b serotype, whereas the serotype 1/2a has been linked to sporadic cases (97). According to other subtyping and phylogenetic analyses, *L. monocytogenes* isolates are classified into 4 distinct lineages (lineage I, II, III, and IV) (98). However, lineage III and IV isolates (mainly serotypes 4a and 4c) are ordinary under-represented, conceivably because of their different phenotypic properties and attenuated pathogenic potential compared to lineage I and II isolates (96, 98). The larger part of human infections are associated to lineage I serotype 4b and 1/2b isolates, despite the fact that lineage II serotype 1/2a isolates have caused some outbreaks (98). According to the temporal and geographical distribution of outbreaks, *L. monocytogenes *has been additionally grouped into epidemic clones (ECs) I (lineage I), II (lineage II), III (lineage I), and IV (lineage I). This differentiation enables discrimination of isolates from various serotypes in view of their ecological compartments (98). Three highly clonal lineage I serotype 4b strains have caused recurrent worldwide outbreaks (ECs I, Ia, and II) (99). 

Moreover, characterization of Listeria spp. depends on genotype characterization, ideally should be based on detection of bacterial virulence genes or gene products. Therefore, a number of important Listeria genes, which are outstanding as important virulence factors in pathogenicity of *L. monocytogenes*, include *plcB* (encoding phospholipase C), *hly* (listeriolysin O), internalin (encoded by *inlA, inlB, inlC,* and *inlJ* genes), *prfA* (transcriptional regulatory protein), *mpl* (metalloprotease), *actA* (Actin assembly-inducing protein A) and *iap* (Invasion associated protein) (62, 100). 

Determining the route of infection requires the discrimination of the bacteria at the strain level, and molecular typing methods are consequently utilized in many cases. Fragment analysis methods such as pulsed-field gel electrophoresis (PFGE), ribotyping and amplified fragment length polymorphism (AFLP), are frequently used to discriminate between strains of *L. monocytogenes* (11). However, because of subtle variations in the concentration and mobility of the bands in these methods, it is hard to communicate the data at a global level (101). Also, these fragment analysis strategies have the drawback (102). Thus, strain discrimination strategies utilizing DNA sequencing techniques have become commonplace in recent years, and methods have been developed in which sequence analysis is performed in several regions of the genome, such that strains are discerned from comparison of the base sequences. Such methods include multilocus variable number of tandem repeat analysis (MLVA) and multilocus sequence typing (MLST) (103-105). In addition, because there are several reports with respect to the resistance of *L. monocytogenes* to antibiotic agents that are specially involved in listeriosis treatment, exhibiting the existence of resistance in *L. monocytogenes* isolated from various sources (food, clinical, animal and environmental) is a major concern (106). Recently, whole genome sequencing (WGS) based analyses have showed a valuable potential in identification of novel gene/features and pathogen fingerprinting associated with specific phenotypes. WGS innovation has enhanced foodborne disease epidemiology of *L. monocytogenes* (107), including tracing a listeriosis outbreak back to a food processing facility source (108). Therefore, the advent of WGS has provided the scientific world with a plethora of methods for further identification and characterization of foodborne pathogens. However, caution is needed when determining which of these methods to use and when they would be useful.

## Conclusion

This review has been shown the presence of Listeria spp. in a variety of raw and RTE food samples in Iran. These products are well contaminated with Listeria spp. and particularly *L. monocytogenes*. Cross contamination from infected staffs, contact with intestinal substance, manipulation and inappropriate transportation and also using contaminated equipments are the fundamental factors for food contamination. Additionally, maybe some food safety and quality standards need to be applied and performed during preparation, transmission, distribution and storage periods. The appropriate cocking of foods can decrease the microbial loads of these products particularly for Listeria species. However, reports of listeriosis from humans in Iran are unclear, either due to losing on recognizing the isolates, unsuitable isolation techniques or absence of awareness. Therefore, further surveillance of the prevalence of *L. monocytogenes* and also of emerging antibiotic resistance is required to enable the recognition of the contaminated foods, as well as ensures the effective antibiotic treatment. In addition, the new researches such as the influence of microbiomes and degree of immune suppression in immunocompromised patients are important topics that require further researches. 

## Conflicts of Interest

We declare no conflict of interest for the authors of the present study.
